# Risk factors for hospital readmission among Swedish older adults

**DOI:** 10.1007/s41999-018-0101-z

**Published:** 2018-09-06

**Authors:** Jenny Hallgren, Anna K. Dahl Aslan

**Affiliations:** 1 Institute of Gerontology, School of Health and Welfare, Jönköping University, 551 11 Jönköping, Sweden; 2 Department of Medical Epidemiology and Biostatistics, Karolinska Institutet, Stockholm, Sweden

**Keywords:** Readmission, Prospective design, Older persons, Falls, Logistic regression

## Abstract

**Introduction:**

Hospital readmissions of older persons are common and often associated with complex health problems. The objectives were to analyze risk factors for readmission within 30 days from hospital discharge.

**Methods:**

A prospective study with a multifactorial approach based on the population-based longitudinal Swedish Adoption/Twin Study of Aging (SATSA) was conducted. During 9 years of follow-up, information on hospitalizations, readmissions and associated diagnoses were obtained from national registers. Logistic regression models controlling for age and sex were conducted to analyze risk factors for readmissions.

**Results:**

Of the 772 participants, [mean age 69.7 (± 11.1), 84 (63%)] were hospitalized and among these 208 (43%) had one or several readmissions within 30 days during the follow-up period. Most of the readmissions (57%) occurred within the first week; mean days from hospital discharge to readmission was 7.9 (± 6.2). The most common causes of admission and readmission were cardiovascular diseases and tumors. Only 8% of the readmissions were regarded as avoidable admissions. In a multivariate logistic regression, falling within the last 12 months (OR 0.57, *p* = 0.039) and being a male (OR 1.84, *p* = 0.006) increased the risk of readmission.

**Conclusions:**

Most older persons that are readmitted return to hospital within the first week after discharge. Experiencing a fall was a particular risk factor of readmission. Preventive actions should preferably take place already at the hospital to reduce the numbers of readmission. Still, it should be remembered that most readmissions were considered to be necessary.

## Introduction

Older persons often require hospitalizations. In many countries the length of stays in hospitals has decreased during the past decades, consequently many patients are poorer at discharge, and readmissions are common [1, 2]. About 30% of the readmissions are believed to be preventable [3]. Hence, identifying which patients that are at risk for readmission is important, as hospitalizations are associated with an increased risk of iatrogenic disorders, confusion and falls that may involve unnecessary suffering [4].

Many studies have described risk factors of readmission as well as risk prediction tools [5]. However, many risk prediction models have poor predictive ability, mostly since readmission risk prediction is complex. Potential risk factors for readmissions seen in earlier research are higher age, numbers of drugs, length of hospital stay, and functional impairment [6–9], but also social factors such as living alone and dissatisfaction with primary care physicians [10]. In addition, older persons seem to have a greater trust in hospital care as compared to primary care and home health care [11, 12], affecting the propensity to seek hospital care. As one third of the readmissions are made without prior medical consultation [13], research about readmissions needs to be viewed from non- medical angles. In this study, we used a population-based prospective study to analyze a wide variety of risk factors of readmissions. The objectives were to analyze risk factors for readmission within 30 days from hospital discharge.

## Methods

### Study population and data sources

We used data from the population-based longitudinal study Swedish Adoption/Twin Study of Ageing (SATSA). The participants in SATSA were drawn from the Swedish Twin Registries (STR) and included same-sex twin pairs reared together and same-sex twin pairs reared apart. The selection criteria have been described in detail previously [14]. In brief, SATSA started in 1984 when the first Questionnaire (Q1) was sent out with the aim to study etiology of individual differences in ageing. SATSA included questions related to self-rated health, loneliness, depression, personality, medications, social networks and housing situation. The fifth questionnaire (Q5) wave in SATSA, which was sent out in 2003, included participants in ordinary housing (*N* = 772) and provides the baseline in this study. The study sample was created by linking data from SATSA to the registry data from The Swedish National Inpatient Register (NPR). The participants were prospectively followed from the baseline survey in 2003 to their first hospitalization after baseline and to their next hospitalization within 30 days, or to the end of the follow-up in the study (31 Dec 2012). In Sweden, hospital care is mainly tax-funded that ensures everyone equal access to health care services. All hospitalization events in this study was defined as a hospital admission due to any cause that included an overnight stay as recorded in the NPR. The main diagnosis of the admissions was registered according to the World Health Organization’s ICD-10 (International Classification of Diseases). Diagnoses were categorized into groups in accordance with the sections of the ICD-10.

### Outcome

The primary outcome in this study was hospital readmission, defined as any hospital readmission within 30 days of discharge of an admission from any cause in the same participant. We excluded readmissions that could involve transfer to another acute care facility before discharge.

### Risk factors

Risk factors for readmissions within 30 days were identified from the literature [5]. Demographic factors included i.e., age, sex, marital status, level of education [dichotomized as upper secondary or university education (1) and compulsory or vocational education (0)], objective and subjective socioeconomic status (SES), as well as childhood SES (high score implies higher SES).

In addition, we included social support and personality, factors that have not previously been widely studied in association with readmissions. Locus of control included three subscales: sense of personal control or lack of control over the direction of one’s own life (Life Direction), beliefs about how responsible people are for misfortunes in their lives (Responsibility), and beliefs concerning the role of luck in determining people’s outcomes (Luck) [15]. Personality traits included neuroticism and extraversion from the EPI [16], EAS temperaments: activity, emotionality, sociability and fear [17], impulsivity [18], modified openness to experience [19], Type-A behavior hard driving [20] and paranoid hostility and cynicism [21] as well as optimism and pessimism scales as modified by Plomin et al. [22]. Whether the participants were troubled by feelings of loneliness or not was dichotomized as always/often (1) versus never/seldom (0).

Health factors included both objective and subjective health. Objective health was indexed as the number of up to 13 organ systems affected by disease. Subjective health included life satisfaction [23], and a self-rated health composite scale (general health now, health now vs. 3 years ago, own health compared with that of others, activities limited by health). Mental health was included using the Center for Epidemiologic Studies Depression Scale (CES-D) [24]. We included the participants’ smoking status (Nonsmoker, Ex-smoker or Current smoker), and level of physical activity was dichotomized as active (daily or once/a couple of times weekly) (1) or inactive (less than weekly) (0). Functional status was included as Activities of Daily Living (ADL) with a maximum score of 10 indicating impairment in all ADLs, and a score of 0 indicating a completely independent individual. We also included self-reported incidence of falls in the past year dichotomized as whether the participant had fallen and landed on the floor (1) versus not (0).

Social support was measured with social support scales; friends support, relatives support and perceived support [25]. Whether a participant regularly (at least once a week) received help or was looked after by an immediate family member, relatives, social worker or health staff was dichotomized as (1), or not (0).

### Statistical analyses

The χ^2^ test or *t* test was used for comparison between groups of individuals who had both been hospitalized, one group experienced a readmission (within 30 days) and the other group did not. For descriptive purposes, numbers of Ambulatory Care Sensitive Conditions (ACSC) among the readmissions, numbers of readmissions during the participation in the study and length of stay in hospital, were also described.

The relationship between readmission, that is, the participants’ first hospital readmission within 30 days of discharge of an admission from any cause, and potential risk factors were analyzed using a bivariate logistic regression model, controlling for age at hospitalization and sex. Risk factors considered as significant (*p* < 0.05) from the bivariate logistic regression model, were entered simultaneously in a multivariable model, controlling for age and sex. To allow for easier comparisons, continuous variables were standardized using z transformation which have a mean of 0 and a standard deviation of 1. All data were analyzed using StataIC 12.0 and/or SPSS Statistics 21.

## Results

**Fig. 1:**
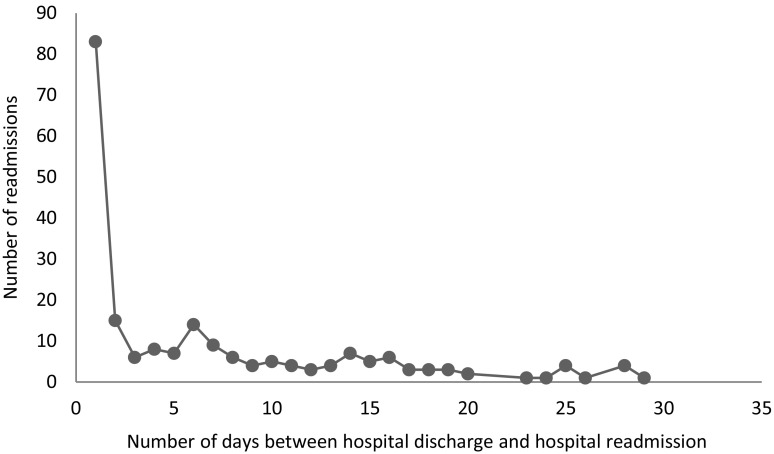
Distribution of days between hospital discharge and hospital readmission among the 553 readmissions

Among the 772 participants, (mean age 69.7 (± 11.1) 59.8% female), 484 (63%) were hospitalized during the study period and among those, 208 (43%) had one or several readmissions within 30 days, for a total of 553 readmissions. The vast majority was readmitted within the first week (Fig. 1). Baseline characteristics and differences between the participants that were readmitted and the participants experiencing an occasional hospitalization are provided in Table 1. The mean age in the readmitted group was 72.6 (± 10.6), and 70.0 (± 10.5) years in the occasional hospitalization group. The mean number of days from hospitalization to readmission was 7.92 (± 6.2, range 1–30). The mean length of stay for hospitalizations that later ended up in a readmission was 8.3, and the mean length of stay during the readmissions was 10.6 days.

**Table 1:** Baseline characteristics and differences between hospitalized and not hospitalized participants, and differences between participants with occasional hospitalizations or readmissions

	Hospitalized *n* = 484	Not hospitalized *n* = 288	*p*	Hospitalized occasional *n* = 276	Hospitalized readmitted *n* = 208	*p*
Demographic characteristic						
Age (45.6–102.7), mean (sd)	71.5 (10.7)	66.7 (11.2)	< 0.001	70.0 (10.5)	72.6 (10.6)	< 0.001
Sex (male), *n* (%)	202 (41.7)	108 (37.5)	0.256	106 (38.4)	96 (46.2)	0.087
Never married, *n* (%)	32 (6.7)	34 (11.9)	0.016	17 (6.2)	15 (7.4)	
Married/cohabiting, *n* (%)	301 (63.1)	169 (59.1)	0.360	184 (67.4)	117 (57.4)	
Widow/widower, *n* (%)	82 (17.2)	51 (17.8)	0.844	39 (14.3)	43 (21.1)	0.131
Divorced, *n* (%)	62 (13.0)	32 (11.2)	0.570	33 (12.1)	29 (14.2)	
Education (Upper Secondary/University vs Compulsory/Vocational), *n* (%)	80 (17.1)	63 (24.3)	0.020	53 (19.8)	27 (13.6)	0.078
Locus of control						
Life direction (5–20), mean (sd)^a^	13.7 (2.6)	13.6 (2.7)	0.015	13.4 (2.6)	12.8 (2.6)	0.017
Responsibility (4–20), mean (sd)^a^	12.0 (3.2)	11.6 (3.1)	0.057	11.6 (3.1)	12.6 (3.2)	0.001
Luck (3–15), mean (sd)^a^	8.8 (2.3)	9.3 (2.3)	< 0.001	8.9 (2.3)	8.5 (2.2)	0.015
Personality						
Neuroticism (0–9), mean (sd)^a^	2.5 (2.1)	2.2 (2.1)	0.156	2.3 (1.9)	2.6 (2.3)	0.140
Extraversion (0–9), mean (sd)^a^	5.2 (2.2)	5.2 (2.4)	0.996	5.1 (2.1)	5.3 (2.3)	0.560
Active (1–5), mean (sd)^a^	2.8 (0.9)	2.9 (0.8)	0.059	2.8 (0.8)	2.7 (0.8)	0.201
Emotionality (1–5), man (sd)^a^	2.9 (0.7)	2.8 (0.6)	0.405	2.8 (0.6)	2.9 (0.7)	0.237
Sociability (1.3–5), mean (sd)^a^	3.7 (0.7)	3.7 (0.6)	0.119	3.6 (0.7)	3.7 (0.7)	0.649
Fear (1–4.8), mean (sd)^a^	2.3 (0.7)	2.3 (0.7)	0.819	2.3 (0.7)	2.4 (0.8)	0.431
Impulsivity (11–47.8), mean (sd)^a^	26.2 (5.9)	26.1 (5.5)	0.850	26.0 (6.0)	26.6 (5.7)	0.287
Openness (6–30), mean (sd)^a^	18.0 (4.2)	18.2 (4.3)	0.529	18.1 (3.9)	17.9 (4.5)	0.701
Hard driving (5–21), mean (sd)^a^	11.4 (3.1)	12.0 (3.2)	0.021	11.6 (3.2)	11.2 (3.1)	0.219
Paranoid hostility (5–23), mean (sd)^a^	10.8 (3.5)	10.6 (3.5)	0.526	10.7 (3.5)	10.9 (3.5)	0.487
Cynicism (5–25), mean (sd)^a^	12.6 (3.8)	12.1 (3.7)	0.061	12.3 (3.6)	13.0 (3.9)	0.044
Optimism scale (6–20), mean (sd)^a^	14.8 (2.4)	14.9 (2.3)	0.562	14.9 (2.4)	14.7 (2.3)	0.218
Pessimism scale (4–20), mean (sd)^a^	9.9 (3.1)	9.5 (3.1)	0.035	9.7 (2.9)	10.3 (3.2)	0.029
Socioeconomic situation						
SES during childhood (− 7.3–17.1), mean (sd)^a^	0.5 (3.9)	1.1 (4.1)	0.086	0.5 (3.8)	0.6 (4.1)	0.724
Subjective SES (− 11.3–7.9) (high score = high SES), mean (sd)^a^	− 0.0 (2.8)	0.3 (2.6)	0.227	− 0.1 (2.9)	0.1 (2.6)	0.405
Objective SES (− 11.3–7.9) (high score = high SES), mean (sd)^a^	0.6 (2.1)	0.9 (2.1)	0.116	0.8 (2.0)	0.3 (2.2)	0.017
Social network						
Regularly receive help or are looked after (yes), *n* (%)	61 (12.7)	18 (6.2)	0.005	24 (8.8)	37 (17.9)	0.003
Support from relatives (− 9.4–3.9), mean (sd)^a^	0.2 (3.0)	− 0.3 (3.1)	0.032	0.1 (3.2)	0.5 (2.9)	0.145
Percieved support (12–30), mean (sd)^a^	23.3 (2.1)	23.2 (1.9)	0.751	23.3 (2.1)	23.3 (2.0)	0.964
Support from friends (− 16.8–8.1), mean (sd)^a^	− 0.1 (5.8)	0.9 (5.0)	0.015	− 0.3 (5.7)	0.1 (5.9)	0.461
Troubled by feelings of loneliness (yes), *n* (%)	66 (13.9)	29 (10.2)	0.142	28 (10.3)	38 (18.7)	0.009
Health characteristics						
Number of illnesses (0–11), mean (sd)^a^	3.4 (2.3)	2.7 (2.0)	< 0.001	3.1 (2.2)	3.9 (2.4)	< 0.001
Depressed mood (0–50), mean (sd)^a^	12.8 (8.5)	11.6 (8.3)	0.071	12.2 (8.2)	13.6 (8.7)	0.078
Self-rated health (− 9.1–5.2), mean (sd)^a^	− 0.6 (3.2)	0.3 (2.8)	<0.001	− 0.1 (3.0)	− 1.3 (3.4)	< 0.001
Life satisfaction (19–63), mean (sd)^a^	44.8 (7.3)	45.3 (7.2)	0.387	45.6 (7.0)	43.8 (7.6)	0.007
Fall in the last 12 month (yes), *n* (%)	93 (20.0)	31 (10.9)	< 0.001	38 (14.6)	55 (27.0)	0.001
Physically active (yes), *n* (%)	81 (18.7)	68 (26.1)	0.028	49 (19.8)	32 (17.1)	0.534
Activity of daily living (0–10), mean (sd)	0.27 (0.74)	0.33 (1.23)	0.410	0.24 (0.68)	0.31 (0.82)	0.281
Smoking status						
Nonsmoker, *n* (%)	368 (80.2)	212 (76.8)	0.452	206 (79.5)	162 (81.0)	
Ex-smoker, *n* (%)	14 (3.1)	10 (3.6)	0.654	5 (1.9)	9 (4.5)	0.168
Current smoker, *n* (%)	77 (16.8)	54 (19.6)	0.309	48 (18.5)	29 (14.5)	
Hospitalization characteristics						
Numbers of admissions, mean (sd)	2.0 (0.8)				2.7 (0.5)	
Number of readmissions, mean (sd)					1.1 (1.9)	
Length of stay, mean (sd)	5.3 (9.6)			4.6 (5.2)	6.2 (13.2)	0.076
Number of days between first and subsequent readmission, mean (sd)					7.8 (7.0)	

^a^Based on scales

The most common causes for the hospitalizations that ended up in readmissions, as well as the occasional hospitalizations, were cardiovascular diseases and tumors (Table 2). Among the 553 readmissions, 44 (8.0%) were regarded as ACSC. Higher age, lower life direction and luck, higher responsibility (beliefs about how responsible people are for misfortunes in their lives), cynicism and pessimism, lower objective SES, receiving more help and more often troubled by feelings of loneliness, greater number of illnesses, lower self-rated health and life satisfaction and experiencing more falls were more common among the readmitted participants (Table 1).

**Table 2:** Primary diagnoses from the occasional hospitalizations and hospitalizations that ended up in readmissions

Primary diagnoses	Hospitalization occasional *n* (%)	Hospitalized readmitted *n* (%)
Cardiovascular diseases	132 (24.0)	126 (22.8)
Tumors	93 (16.9)	70 (12.7)
Injuries, fractures	35 (6.4)	52 (9.8)
Diagnoses of symptoms	42 (7.7)	56 (10.1)
Diseases of the respiratory tract including pneumonia	52 (9.5)	49 (8.9)
Gastrointestinal diseases	37 (6.7)	34 (6.1)
Muscle and joint diseases	27 (4.9)	29 (5.2)
Diseases of the urogenital tract	27 (4.9)	31 (5.6)
Endocrinological diseases	7 (1.3)	7 (1.3)
Others	28 (5.1)	54 (9.8)
Psychiatric disorders including dementia	22 (4.0)	17 (3.1)
Neurological diseases	17 (3.1)	12 (2.2)
Infections	13 (2.4)	3 (0.5)
Diseases of the sense organs	5 (0.9)	5 (0.9)
Skin diseases	4 (0.7)	5 (0.9)
Total	549	550

The bivariate logistic regression revealed that higher age, male sex, responsibility, feelings of loneliness, number of illnesses, self-rated health, life-satisfaction, falling in the last 12 months were significantly associated with an increased risk of readmission (Table 3).

**Table 3:** Bivariate logistic regression model of readmissions risk controlled for age and sex

	All *N*			
	*B* (SE)	*p*	Odds ratio	95% CI
Demographic characteristic				
Age	0.034 (0.009)	< 0.001	1.035	1.02–2.11
Sex (male)	0.376 (0.190)	0.047	1.457	1.00–2.11
Married/cohabitating (ref)	1	1	1	1
Never married	− 0.445 (0.288)	0.123	0.641	0.364–1.128
Widow/widower	− 0.143 (0.445)	0.747	0.867	0.362–2.072
Divorced	− 0.124 (0.369)	0.738	0.884	0.429–1.822
Education (Upper secondary/University)	0.310 (0.269)	0.248	1.364	0.806–2.310
Education (compulsory/vocational)	1		1	
Locus of control				
Life Direction^a^	− 0.065 (0.038)	0.087	0.937	0.871–1.010
Responsibility^a^	0.074 (0.032)	0.023	1.077	1.010–1.147
Luck^a^	− 0.057 (0.045)	0.213	0.945	0.865–1.033
Personality				
Neuroticism^a^	0.050 (0.046)	0.278	1.051	0.961–1.149
Extraversion^a^	0.029 (0.044)	0.506	1.030	0.944–1.123
Active^a^	− 0.102 (0.112)	0.362	0.903	0.725–1.125
Emotionality^a^	0.041 (0.145)	0.777	1.042	0.785–1.384
Sociability^a^	0.099 (0.145)	0.496	1.104	0.831–1.465
Fear^a^	0.122 (0.137)	0.374	1.129	0.864–1.477
Impulsivity^a^	0.019 (0.016)	0.252	1.019	0.987–1.052
Openness^a^	0.006 (0.023)	0.798	1.006	0.961–1.053
Hard driving^a^	− 0.028 (0.031)	0.372	0.973	0.915–1.034
Paranoid Hostility^a^	0.005 (0.028)	0.845	1.005	0.952–1.062
Cynicism^a^	0.029 (0.026)	0.271	1.029	0.978–1.084
Optimism scale, positive items^a^	− 0.057 (0.040)	0.160	0.945	0.873–1.023
Pessimism scale, negative items^a^	0.038 (0.034)	0.252	1.039	0.973–1.110
Socio economic situation				
SES during childhood^a^	0.021 (0.043)	0.617	1.022	0.940–1.111
Subjective SES^a^	0.017 (0.035)	0.618	1.018	0.950–1.090
Objective SES^a^	− 0.059 (0.051)	0.242	0.943	0.854–1.041
Social network				
Regularly receive help or are looked after (yes)	− 0.472 (0.308)	0.126	0.624	0.341–1.141
Regularly receive help or are looked after (no)	1		1	
Support from friends^a^	0.022 (0.017)	0.206	1.022	0.988–1.058
Perceived support^a^	− 0.007 (0.050)	0.892	0.993	0.900–1.096
Support from relatives^a^	0.047 (0.032)	0.145	1.048	0.984–1.117
Troubled by feelings of loneliness? (yes)	− 0.631 (0.279)	0.024	0.532	0.308–0.918
Troubled by feelings of loneliness? (no)	1	1	1	1
Health characteristics				
Number of illnesses	0.133 (0.045)	0.003	1.143	1.046–1.249
Depressed mood^a^	0.058 (0.032)	0.069	1.060	0.996–1.128
Self-rated health^a^	− 0.106 (0.031)	0.001	0.899	0.846–0.956
Life satisfaction^a^	− 0.033 (0.013)	0.015	0.968	0.943–0.994
Fall in the last 12 month (yes)	− 0.699 (0.246	0.005	0.497	0.307–0.805
Fall in the last 12 month (no)	1		1	
Physically active (yes)	0.046 (0.260)	0.860	1.047	0.629–1.742
Physically active (no)	1	1	1	1
Activity of daily living	0.023 (0.098)	0.814	1.023	0.844–1.241
Smoking status				
Nonsmoker (ref)	1	1	1	1
Ex-smoker	− 0.042 (0.276)	0.878	0.958	0.558–1.647
Current smoker	0.724 (0.627)	0.249	2.062	0.603–7.047
Hospitalization characteristics				
Number of admissions	2.601 (0.218)	< 0.0001	13.471	8.780–20.668
Length of stay	0.225 (0.140)	0.108	1.252	0.952–1.647

^a^Linear representation. Continuous variables were standardized using *Z* transformation

Factors significantly associated with readmission risk from the bivariate model were entered simultaneously in a multivariate logistic regression model. The results revealed that male sex and falling within the last 12 months increased the risk of readmission. When stratifying the multivariable model on sex, we found that increased numbers of diseases increased the readmission risk for women, but not for men. For women we also noticed a tendency (*p* = 0.059) for lower life satisfaction to be related to increased readmission risk (Table 4).

**Table 4:** Multivariate logistic regression of readmission risk, controlled for age and sex

	All (*N* = 484)				Male (*n* = 202)			Female (*n* = 282)		
	*B* (SE)	*p*	Odds ratio	95% CI	*B* (SE)	*p*	Odds ratio	*B* (SE)	*p*	Odds ratio
Age	0.191 (0.119)	0.108	1.211	0.959–1.530	0.195 (0.193)	0.314	1.215	0.206 (0.158)	0.194	1.675
Sex (male)	0.612 (0.221)	0.006	1.843	1.194–2.845						
Responsibility	0.159 (0.111)	0.154	1.172	0.942–1.448	0.063 (0.166)	0.703	1.065	0.266 (0.155)	0.085	1.768
Feelings of loneliness (yes)	− 0.320 (0.342)	0.350	0.726	0.371–1.421	− 0.122 (0.618)	0.843	0.885	− 0.242 (0.430)	0.785	1.824
Number of illnesses	0.201 (0.120)	0.094	1.223	0.967–1.547	− 0.125 (0.196)	0.594	0.901	0.462 (0.161)	0.004	2.176
Self-rated health	− 0.205 (0.128)	0.109	0.815	0.634–1.047	− 0.233 (0.186)	0.209	0.792	− 0.182 (0.183)	0.321	1.194
Life satisfaction	− 0.136 (0.121)	0.259	0.872	0.688–1.101	0.055 (0.187)	0.767	1.057	− 0.320 (0.167)	0.056	1.008
Fall in the last 12 month (yes)	− 0.558 (0.218)	0.039	0.573	0.329–0.997	− 0.684 (0.487)	0.161	0.505	− 0.509 (0.366)	0.164	1.230

*R*^2^  = 0.110 (Cox & Snell), *σ* 147 (Nagelkerke), Model χ^2^ = 44.810, *p* = < 0.001

## Discussion

In this study, we included a wide range of variables to explore risk factors of readmission. We found that both physical and subjective health issues were related to readmission risk in the bivariate logistic model. In the multivariate model when all risk factors were controlled for, falling within the last 12 months and male sex were significantly associated with increased readmission risk.

We found that the majority of readmissions occurred within the first week after discharge, supporting previous studies [26, 27]. Hospitalization and bedrest itself may lead to decline in ADL and loss of independence [28]. It is possible that clinical preventive actions, and especially for persons with a history of falls, should be most effective if targeting the first week after discharge and/or preparing the patients with proper care planning already at the hospital. Reasons for readmissions may vary, but may include a lack of information at discharge from hospital [29], possibly since it has been shown that older patients seldom participate in medical decision making regarding discharge planning [30].

As for many other health outcomes in late life, history of falls was an in important risk factor of being readmitted to the hospital. Even though falls might have multifactorial causes, fall prevention for older persons in general and for older persons that have been hospitalized is important since fall complications may lead to injuries and deaths. Recent studies have shown that more than a third of older adults with a fall-related emergency department (ED) diagnosis, had an ED revisit or died within 1 year [31, 32]. Swedish home care, home health care and hospitals are working preventively and use quality registers to detect risk of falls and plan for preventive actions, nevertheless, falls are common among older adults [33]. The findings in this study highlight the importance of fall prevention and need of extra support and care after discharge.

Although previous studies have shown that personality and social factors were related to readmission risk, [34] the current analyses did not support that conclusion. It is possible that the pathways to readmission differ as for example the health care staff has more information about the patient at the second admission. Another possibility is that care in lower levels was not satisfactory and that the older persons instead decided to transfer to a hospital [12]. On the other hand, only 8% of the readmissions were regarded as avoidable admissions (ACSC) according to the definition [35]; 92% required treatment at the hospital. Hence, a possible interpretation is that these patients might have been discharged too early from the hospital. Length of stay may be an important marker of readmission risk as it has been shown that longer stay in hospital is associated with a decreased risk of readmission [26]. This result is supported by our findings, where those with readmissions had a shorter stay than those that were not readmitted, although length of stay was not significantly associated with readmission risk when other factors were controlled for.

It is possible that the readmission risk between diagnoses varies [27, 36]. In this study, the most common primary diagnoses were cardiovascular diseases, both among the readmissions and the occasional hospitalizations. Chopra and colleagues [36] found that persons with hospitalization due to cardiovascular diseases, diabetes and mental health conditions were more likely to have readmission within 30 days, compared to other conditions.

Further, we found male sex to be associated with readmission risk in the multivariate model, in line with a previous study [37] showing that men had increased readmission risk when treated for pneumonia. In contrary, they also [37] found that women had an increased readmission risk of overall admission causes. In this study, more illnesses were associated with increased readmission risk for women, but not for men. Studies taking gender differences into account are warranted.

There was no difference in ADL between readmitted and occasional hospitalization participants affecting the readmission risk, contrary to what other studies have found [7, 38]. The difference could be explained by disparities in measurements of functional status [38], or by different selections of participants [7]. It could also be explained by the fact that ADLs, as with all the risk factors, were measured at baseline, and thus the analyses did not take into account important events that may have occurred during follow-up. On the other hand, 42% of hospitalizations occurred within 1 year of baseline, and 87% occurred within 4 years. Although this study included several potential risk factors of readmission, we did not have information on cognitive impairment nor information on nutritional status. Another limitation in this study is that it was not possible to control for proximity to hospital, which might have had an impact on the propensity to seek hospital care and on readmissions. This study also had several strengths, including the prospective follow-up design, the population-based sample and the fact that SATSA includes persons from across Sweden with a variety of medical conditions.

## Conclusion

Falling within the last 12 months was associated with readmission risk in a population based prospective follow-up study. Most of the readmissions occurred within the first week. These results suggest that the first week after discharge as well as fall prevention among older persons regarding readmission risk are important. Clinical practice needs to focus on coordinated care post hospitalization to reduce readmission risk.
